# Salivary Chromium and Cobalt Concentrations in Patients with Dental Metallic Restorations—A Pilot Study

**DOI:** 10.3390/dj12110362

**Published:** 2024-11-14

**Authors:** Zlatina Tomova, Desislav Tomov, Delyana Davcheva, Yordanka Uzunova

**Affiliations:** 1 Department of Prosthetic Dental Medicine, Faculty of Dental Medicine, Medical University of Plovdiv, 3, Hristo Botev Blvd., 4002 Plovdiv, Bulgaria; 2 Research Institute at Medical University of Plovdiv, Medical University of Plovdiv, 15-A “Vasil Aprilov” Blvd., 4002 Plovdiv, Bulgaria; desislav.tomov@mu-plovdiv.bg (D.T.); delyana.davcheva@mu-plovdiv.bg (D.D.); 3 Department of Clinical Laboratory, Faculty of Medicine, Medical University of Plovdiv, 15-A “Vasil Aprilov” Blvd., 4002 Plovdiv, Bulgaria; 4 Department of Bioorganic Chemistry, Faculty of Pharmacy, Medical University of Plovdiv, 15-A “Vasil Aprilov” Blvd., 4002 Plovdiv, Bulgaria

**Keywords:** dentistry, dental alloys, ion release, metal ions, saliva

## Abstract

**Introduction:** Metal ions, released from dental alloys due to corrosion, come in contact with the cells of the surrounding tissues and may spread throughout the body via the gastrointestinal system, thus inducing dose-dependent cytopathological effects. This study aimed to assess and compare the salivary cobalt and chromium concentrations in individuals aged 18–65 years with and without dental restorations containing metal alloys. **Methods:** Participants were divided into two main groups according to the existence of metal alloys in the oral cavity—18 patients had fixed prosthetic restorations made of metal alloys, and 17 patients had no metal objects in their oral cavity. Each main group was subdivided into two subgroups according to the type of saliva sample—with or without additional stimulation. Salivary cobalt and chromium concentrations were measured by inductively coupled plasma mass spectrometry. A non-parametric Mann–Whitney test and Spearman’s rank correlation coefficient were applied, and the level of significance was set to *p* < 0.05. **Results**: The results showed that the chromium level in non-stimulated saliva was higher in the group of patients with metal dental restorations. No statistical difference was found in cobalt levels. There was no statistical difference in Co or Cr concentrations in stimulated saliva between the studied groups. A positive correlation was found between Cr and Co concentrations in non-stimulated saliva and between cobalt concentrations in stimulated and non-stimulated saliva. **Conclusions**: Metal alloys in the oral cavity induced elevated chromium levels in non-stimulated saliva, and a correlation between chromium and cobalt ion concentration was found. A detailed examination of patients and their medical history prior to prosthetic treatment is advisable in order to avoid any undesired health effects.

## 1. Introduction

Different types and combinations of artificial materials are used for rehabilitation of defects of dental arches and hard dental tissues. The longevity, esthetics, and safe use of the final dental device depend on the properties of the materials used for its fabrication. Fixed and removable prostheses, onlays, implants, and post-and-core restorations are produced fully or partially from noble or base alloys with different compositions. Although contemporary CAD/CAM technologies allow fast production of all-ceramic restorations, metal ceramic crowns and bridges with a framework of cobalt–chromium and nickel–chromium base dental alloys are still widely used due to their acceptable combination of desired mechanical strength, esthetics, and favorable price [1].

The tendency to corrosion is the major factor defining the biological safety of a dental alloy [2]. Various factors of the surrounding environment initiate the process of deterioration of these metal objects. After placement in the oral cavity, electrochemical corrosion occurs on the metal surface due to the presence of electrolytes. As a result, the released metal ions contact the surrounding tissues and can enter the human body. In addition to electrochemical corrosion, metal ion emission from prosthetic devices may be induced by the mechanical friction of the metal surfaces during mastication, leading to surface deterioration (tribocorrosion) [3]. In the presence of two or more prosthetic restorations fabricated from alloys with different compositions, galvanic corrosion may occur and may change the corrosion properties of the alloys used [4].

The corrosion rate of the metal parts of the dental prosthesis is influenced by the composition of the dental alloy, the method of alloy production and the method of production of the final restoration and may be altered by the specific conditions in the oral cavity [5,6]. Acidity is a factor of major significance for dental alloys—the decrease in pH leads to a decrease in corrosion resistance and in increase in metal ion release [7,8]. Numerous exogenic factors affect the properties of dental alloys used for prosthetic treatment, including intake of nonalcoholic drinks, bleaching agents, fluor-containing gels and mouthwashes, and toothpastes [9]. Fluoride concentration significantly influences the surface morphology and topography of the materials, thus changing the elemental ion release from base dental alloys [10].

Cytopathological effects, which are dose-dependent, may be caused by different types of metal ions [11]. Although chromium (III) is an essential trace element for humans that plays a role in lipid and glucose metabolism, chromium (VI) compounds are toxic and carcinogenic [12,13]. Once ingested orally, chromium (VI) can be reduced to chromium (III) by physiological fluids like saliva and gastric juice and processed by intestinal bacteria. Thus, the extracellular reduction of chromium (VI) is considered a protective mechanism against the cancerogenic effect of the compound. However, a portion of chromium (VI) is able to escape from the reduction process in the gastrointestinal tract and enter human cells, where its transformation to chromium (V), (IV), and (III) is combined with the production of reactive oxygen species and an increase in the intracellular oxidative stress level [14]. Intracellular chromium compounds react with proteins and DNA. Mutations may occur as a result of DNA adducts forming under the intracellular action of chromium (III) [15]. Cobalt is an important element for human health as it is a part of the vitamin B12 molecule—a compound of significant importance for red blood cell formation and DNA synthesis and regulation. The daily intake of cobalt should be around 2.4 μg, although the usual intake is higher—around 5–40 μg [16]. The studies by Bauer et al. showed that cobalt and chromium ions cause concentration-dependent effects on human chondrocytes [17]. According to Messer et al., exposure to hexavalent chromium may cause morphological changes in human gingival fibroblasts [18]. Akbar et al. came to the conclusion that cobalt and chromium (VI) ions may cause lymphocyte apoptosis, thus altering immune system functions [19]. High doses of cobalt may cause acute toxic reactions or adverse effects in chronic exposure. Cobalt may induce hypoxic-like cell responses and elevated oxidative stress levels, which may lead to a shift in the gene and protein expression and may cause an adverse effect on the T-cell population [20]. The risk of carcinogenicity was the major concern that led to the recent reduction by the European Chemical Agency (ECHA) of the derived no-effect level (DNEL) for cobalt taken via the oral route [21]. Cobalt ions are also neurotoxic and may lead to ototoxicity and vision dysfunctions [22]. Although dental alloys are considered relatively safe, with time they undergo changes due to corrosion and wear, and some of their components might affect the body. The most common local manifestations associated with dental alloys are pigmentations of the oral mucosa, lichenoid lesions, leucoplakia, and mouth-burning sensations. Hypersensitivity reactions such as dermatitis, chronic fatigue, headaches, and polyarthritis are systemic manifestations of adverse effects of dental alloys [2].

Therefore, our aim was to assess and compare the salivary concentration of cobalt and chromium in patients with or without metal alloys in their oral cavity. The research hypotheses were that the metal ion concentration in saliva is not affected by the presence of dental metal objects, and that no correlation exists between the concentrations of the studied metal ions in non-stimulated and stimulated saliva.

## 2. Materials and Methods

The study was conducted with the participation of 35 patients, who were divided into two main groups. The first main group consisted of 18 patients who had dental restorations fully or partially made of metal alloys. The types of restorations found in the patients were amalgam obturations, full metal crowns, partially veneered metal crowns and bridges, and metal ceramic crowns and bridges (Table S1 in Supplementary Material). According to the patients’ medical history, as there is still no unified national database of patients’ dossiers, previous dental treatments were performed more than a year earlier (the period of placement of the existing prosthetic devices varied from 1 to 7 years). The participants who had no metals in the oral cavity were placed in the second group (17 patients). Inclusion criteria for the volunteers for the study were age between 18 and 65 years, absence of acute or chronic disease, non-smoking, no signs of acidic erosion or abrasion due to bruxism, and no intake of chromium-containing supplements. Exclusion criteria were presence of chronic diseases requiring intake of systemic medications, acute diseases at the moment of sample collection, smoking, age under or above the defined range, presence of lesions on the oral mucosa, presence of removable partial dentures and implant supported restorations. Participants had no orthodontic appliances. Informed consent was signed by all the participants in the study. The study followed the standards of the Institutional Ethical Committee of the Medical University of Plovdiv, Bulgaria (Decision № 6/4 July 2024).

After the initial examination, the selected patients were given instructions and asked to come to the dental office the next day for sample collection, which was performed before the start of any dental treatment. To avoid circadian rhythms, saliva samples were gathered between 10.00 A.M. and 12 A.M. at least three hours after breakfast and/or toothbrushing. During that period, patients were allowed to drink only water. Patients were not fasting and were advised to be well-hydrated. Samples of non-stimulated and stimulated saliva were obtained from each participant. Before saliva collection, distilled water was used to rinse the mouth. Samples of non-stimulated saliva were harvested in polyethylene containers by spitting the saliva gathered at the bottom of the oral cavity without the use of any external taste or aromatic stimuli. Samples of stimulated saliva were collected using 100 μL of 2% citric acid applied over the tongue every 30 s [23,24]. The time needed for collection of a 15 mL sample was 15–20 min for non-stimulated saliva and 5 min for stimulated saliva. The samples were analyzed at the Research Institute at the Medical University of Plovdiv. Cobalt and chromium concentrations were measured by inductively coupled plasma mass spectrometry (ICP-MS, Thermo Scientific, iCAP Q, Bremen, Germany).

*Saliva sampling*. After collection, saliva samples were stored at −70 °C until analysis. Ten saliva samples, collected from patients without metal dental restorations, were used for the preparation of a saliva pool for the purposes of the internal quality control procedures. All vessels used in the preanalytical and analytical stages were previously tested for Co and Cr contaminants.

*Chemicals and materials.* The standard solutions used for calibration were prepared from NIST-traceable elemental standards. ICP-MS monoelement Cobalt (Co) 10,000 mg/L and chromium (Cr) 1000 mg/L were purchased from CPAchem Ltd. (Stara Zagora, Bulgaria). Yttrium (Y) standard for ICP-MS 10 mg/L for preparation of internal standard (IS) solution was provided by Merck (Darmstadt, Germany). All solutions were prepared with nitric acid (HNO_3_, suprapur, Fisher Scientific, Loughborough, UK) and ultra-pure water (18.2 M Ωcm).

Aqueous calibration solutions for Co and Cr were prepared in 1% HNO_3_ at concentrations of 0.02, 0.1, 0.2, 1.0, 5.0, and 20.0 µg/L. Y was used as the IS in a concentration of 2 µg/L with the same acid content. Three levels of mineralized saliva pool samples were prepared for the purposes of the internal quality control procedures by spiking with 0.1 µg/L, 5.0 µg/L, and 10 µg/L Co/Cr before dilution.

*Digestion method.* Saliva samples were thawed at room temperature, then vortexed for 10 min and pipetted into a microwave digestion vessel. All saliva samples and blanks were then mineralized, following an in-laboratory, modified acid digestion procedure [25] with an optimized digestion program (1.0 mL sample mixed with 4.0 mL of 1%HNO_3_; maximum temperature 190 °C, ramp time 10 min, hold time 10 min). Reagent blanks were prepared by adding deionized water as a sample. After digestion, all mineralizates were cooled to room temperature and then transferred to 15 mL polypropylene tubes for ICP-MS analysis.

*Instrumentation.* Microwave-assisted acid mineralization was performed by a microwave digestion system with closed vessels (Multiwave GO, Anton Paar, Graz, Austria). The water purification system used was PURELAB Chorus 2 (ELGA Lab Water, High Wycombe, UK). The determination of Co and Cr was carried out by ICP-MS. The mass spectrometer was equipped with a collision cell and a kit for online introduction of the internal standard ((Thermo Fisher Scientific, Bremen, Germany). The detailed operating conditions for ICP-MS were as follows: plasma power: 1550 W; nebulizer gas flow (Ar): 1.03 L/min; auxiliary gas flow (Ar): 0.80 L/min; plasma gas flow (Ar): 14 L/min; collision gas flow (He): 4.62 mL/min; dwell time: 0.3 s; sweeps: 15; and replicates: 3. Kinetic energy discrimination (KED) mode was used to measure the signals of the isotopes (^53^Cr, ^59^Co, ^89^Y). To enable the analysis of ele-ments and isotopes the Thermo Scientific QtegraTM Software 2014 (Thermo Fisher Scientific Inc., Waltham, MA, USA) was used.

Saliva samples were divided into four subgroups:

Group 1.1—non-stimulated saliva (NS) from patients with metal dental restorations (18 samples).

Group 2.1—non-stimulated saliva (NS) from patients without metal dental restorations (17 samples).

Group 1.2—stimulated saliva (SS) from patients with metal dental restorations (18 samples).

Group 2.2—stimulated saliva (SS) from patients without metal dental restorations (17 samples).

SPSS statistical package (version 17.0) was used for statistical data processing. Because of the non-Gaussian distribution of the results, a non-parametric Mann–Whitney test and Spearman’s rank correlation coefficient were applied. The level of significance was set to *p* < 0.05.

## 3. Results

The salivary samples of all participants in the study were analyzed by ICP-MS. In order to ensure analytical reliability of the results, all samples were mineralized and analyzed in parallel with three levels of spiked saliva pool samples (0.1 µg/L, 5.0 µg/L and 10 µg/L Co/Cr).

The descriptive statistics are presented in Table 1 and Table 2.

**Figure 1:**
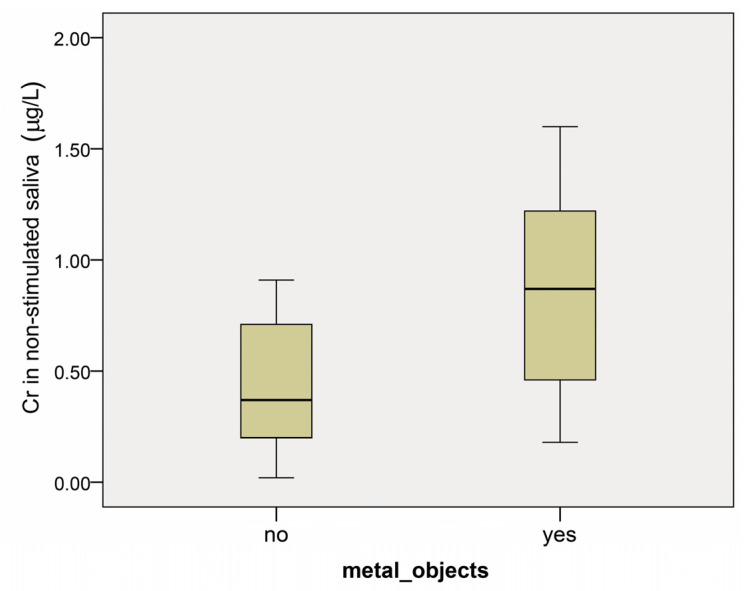
Chromium concentration (µg/L) in non-stimulated saliva in patients with (yes) and without (no) prosthetic restorations before the start of the dental treatment. Results are presented as median, minimum, and maximum values.

The results are presented as median values. The chromium concentration in non-stimulated saliva in the group with metal objects was 0.82 µg/L (95% confidence interval 0.36–1.79) vs. 0.37 µg/L (95% confidence interval 0.29–0.57) in the group without metal objects, showing a statistically significant difference (*p* = 0.017) (Figure 1).

**Figure 2:**
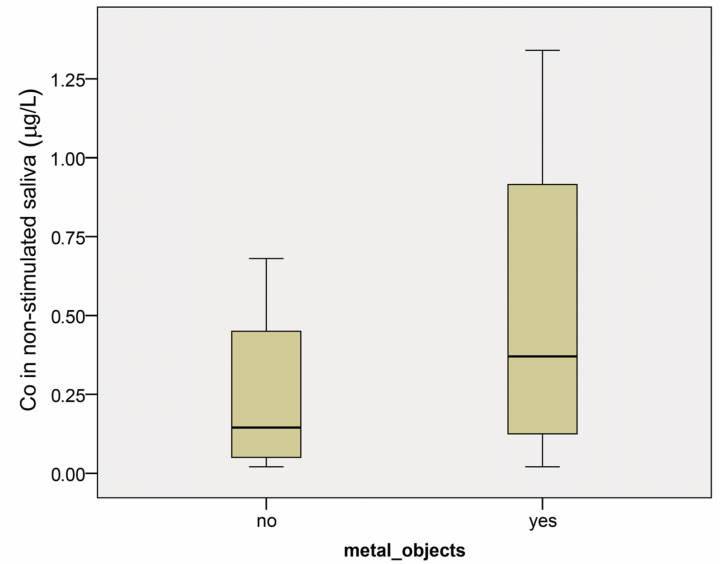
Cobalt concentration (µg/L) in non-stimulated saliva in patients with (yes) and without (no) prosthetic restorations before the start of any dental treatment. Results are presented as medians and minimum and maximum values.

No statistical difference was found in cobalt level in non-stimulated saliva between the groups with and without metal restorations (*p* = 0.363) (Figure 2). The median values with confidence intervals found were 0.15 µg/L (0.04–0.74) and 0.28 µg/L (0.22–1.10) for groups 1.1 and 2.1, respectively.

**Figure 3:**
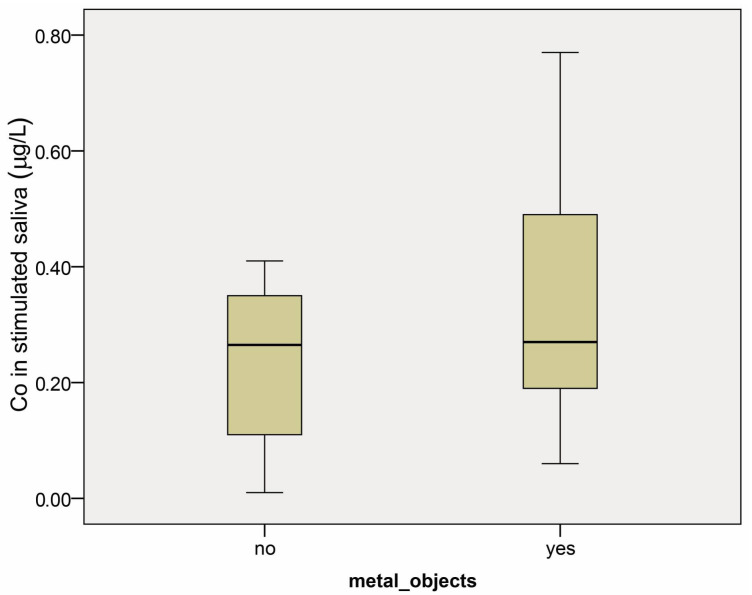
Cobalt concentration (µg/L) in stimulated saliva in patients with (yes) and without (no) prosthetic restorations before the start of any dental treatment. Results are presented as medians and minimum and maximum values.

**Figure 4:**
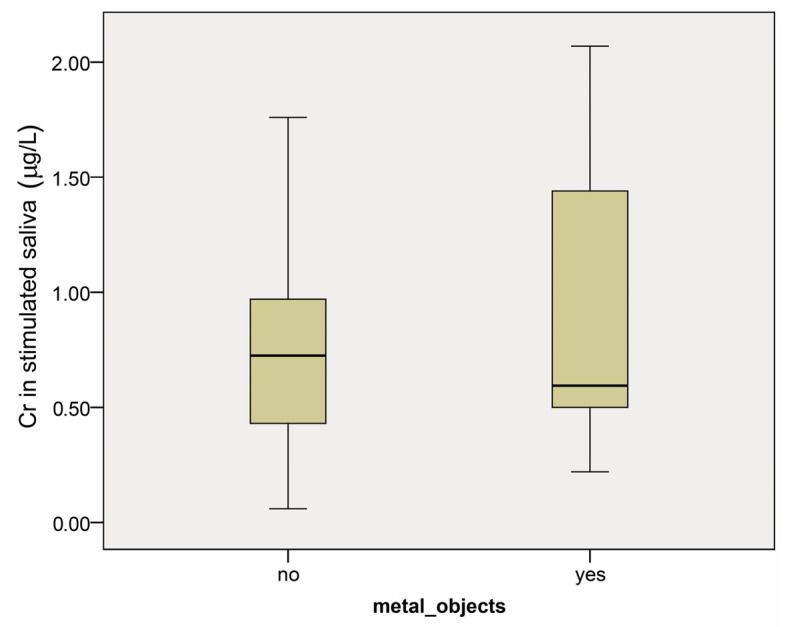
Chromium concentration (µg/L) in stimulated saliva in patients with (yes) and without (no) prosthetic restorations before the start of any dental treatment. Results are presented as medians and minimum and maximum values.

There was no statistical difference in the Co and Cr concentrations in stimulated saliva (SS) between the studied groups (*p* = 0.37 and *p* = 0.843, respectively) (Figure 3 and Figure 4). The median values for cobalt in groups 1.2 and 2.2 were 0.27 µg/L, with confidence intervals (0.22–0.52) and (0.02–1.05), respectively. Chromium median values were 0.59 µg/L (confidence interval 0.52–1.34) and 0.73 µg/L (confidence interval 0.51–0.93) in groups 1.2 and 2.2.

There was a positive correlation between Cr and Co concentration in non-stimulated saliva in group 1.1 (r = 0.551, *p* = 0.022). A statistically significant correlation between the cobalt concentration in non-stimulated (group 1.1) and stimulated saliva (group 1.2) was found (r = 0.624, *p* = 0.01).

## 4. Discussion

The research hypotheses that the metal ion concentration in saliva is not affected by the presence of dental metal objects and that there is no correlation between the concentrations of the studied metal ions in saliva were rejected.

The levels of chromium and cobalt found in the patients not wearing metal restorations suggest the existence of other ways by which these ions can enter the saliva. In their research, Sun et al. claimed that the main source of chromium is food and drinking water [15].

Chromium is an element that is always present in the composition of cobalt–chromium and nickel–chromium dental alloys and its content may vary in a wide range (22–35 wt.%). The chromium on the surface of chromium-containing alloys is rapidly converted to its trivalent oxide, providing resistance to corrosion. Other elements may influence the effectiveness of chromium in forming or maintaining the film, for example nickel promotes re-passivation, especially in reducing environments, and molybdenum stabilizes the passive film in the presence of chlorides [26,27]. These findings lead to the conclusion that the exact composition defines the corrosion resistance of each specific alloy [28]. Surface passivation and oral biofilms limit metal ion emission from metal restorations [29]. Studies by Lu et al. show that some microorganisms might enhance alloy resistance to corrosion [30]. The elevated chromium levels in patients with prosthetic dental restorations may hide a risk of potential adverse effects to the body.

Chromium release has been demonstrated from prosthodontic frameworks and orthodontic appliances [31,32]. Therefore, the presence of this element should be carefully considered.

The higher concentration of chromium ions in non-stimulated saliva in patients with metal prosthetic restorations may be due to emission of chromium because of deterioration of the passive surface layer. Cell-induced, localized types of corrosion attack and wear during sliding (tribocorrosion) may influence the integrity of the oxide layer of dichromium trioxide, thus deviating the corrosion properties of the alloy and increasing the metal ion emission [33]. The absence of a significant difference in cobalt level in non-stimulated saliva between the groups with and without metal presence may be due to the fast recovery of the surface layer of Cr_2_O_3_ in the presence of oxygen in the surrounding medium, which prevents emission of cobalt ions from the alloy [34].

The insignificant differences in cobalt and chromium levels in stimulated saliva between the studied groups may indicate that metal ions released from the dental restorations do not influence distant areas of the body, like salivary glands, which do not directly contact the prosthetic devices. Stimulated saliva comes directly from the salivary glands, and the time it stays in the oral cavity is probably not enough for metal ions eluted from the metal surfaces to cause a significant change in its composition. The insignificant differences in the stimulated saliva subgroups could also be related to the washing effect of excessively secreted saliva.

The positive correlation between chromium and cobalt concentration in non-stimulated saliva samples from patients with metal restorations found in our study suggests that the weaker the passive chromium oxide layer, the higher the potential harmful cobalt ion emission. If the oxide layer is intermittently affected by local factors (transient change in acidity, use of fluor-containing mouthwashes, tribocorrosion, pathogalvanic coupling with other alloys present in the oral cavity, etc.), a risk of cobalt leaching occurs [10]. The positive correlation between the cobalt concentration in non-stimulated and stimulated saliva samples in patients with metal restorations is a matter of concern—it is possible not only local, but potentially general pathological effects are to be expected [35]. In the era of CAD/CAM technologies, new powder dental alloys are developed for 3D printing, and their properties, especially corrosion resistance, are of great significance for the biocompatibility of the final restoration. In cases of prosthetic devices with large, exposed metal surfaces, the influence of the oral conditions may lead to deterioration of a larger part of the chromium passive layer, thus creating a higher risk of cobalt release and chronic exposure. According to EU Medical Devices Regulation (MDR), cobalt is defined as a C1B M2 R1B substance in the CMR classification (carcinogenic, mutagenic, and toxic to reproduction). Due to health concerns, the EU is considering restrictions on the use of cobalt in medical devices if its content exceeds 0.1% [36].

Our results confirm the findings of Garhammer et al., according to which salivary metal ion concentration is influenced, among other things, by intraoral metal restorations [37]. Yassaei et al. found increased salivary chromium concentrations in patients undergoing orthodontic treatment, but the differences with the initial chromium levels were not significant [38].

The small number of patients studied and the lack of clinical trials were some of the limitations of this study. There was no information about the chemical composition of the base alloys used to produce the dental restorations. In the last two decades, the most common base alloys used in Bulgaria for fixed-prosthetic restorations have been not Ni–Cr, but Co–Cr. Unfortunately, there is no national database for patients’ dossiers or official documentation about previously performed dental treatments. This is the main reason why the composition of the main metal alloys used in the production of restorations in recent years has not been clarified. The patients are not aware of the types of materials used for their treatment. By assessing the metal ion release from the old prosthetic restorations, we could possibly recommend their removal and placement of new ones if the results suggest possible risk effects. Pan et al. found that nickel–chromium and cobalt–chromium alloys induce transient trace metal accumulation and apoptotic effects in the liver and kidney, which can be reduced or terminated by the removal of the alloys [39]. As there are no defined reference values for cobalt and chromium in saliva for the Bulgarian population, the results cannot confirm or reject the possible adverse effects of these metal ions on the health status of patients treated with metal dental restorations. Further investigations are needed for confirmation of the correlation between the presence of oral metal restorations and salivary metal ion levels.

## 5. Conclusions

Based on the results of this pilot study and considering its limitations, it can be assumed that metal alloys in the oral cavity induce elevated chromium levels in non-stimulated saliva, and a correlation between chromium and cobalt ion concentrations is found. A detailed examination of patients and their medical history prior to prosthetic treatment is advisable in order to avoid any undesired health effects.

## Figures and Tables

**Table 1:** Descriptive analysis of cobalt concentration (µg/L).

Saliva Sample	Presence of Metal Objects	Min	Max	Median	Mean	St. Dev.
NS	Yes	0.02	2.83	0.28	0.44	0.86
NS	No	0.00	3.45	0.15	0.66	0.81
SS	Yes	0.06	1.18	0.27	0.37	0.28
SS	No	0.01	4.71	0.27	0.55	1.1

**Table 2:** Descriptive analysis of chromium concentration (µg/L).

Saliva Sample	Presence of Metal Objects	Min	Max	Median	Mean	St. Dev.
NS	Yes	0.00	6.21	0.82	1.07	1.39
NS	No	0.02	0.91	0.37	0.43	0.28
SS	Yes	0.00	2.93	0.59	0.93	0.79
SS	No	0.06	1.76	0.73	0.72	0.42

## Data Availability

The original contributions presented in the study are included in the article/Supplementary Material, further inquiries can be directed to the corresponding authors.

## Supplementary Materials

The following supporting information can be downloaded at: https://www.mdpi.com/article/10.3390/dj12110362/s1, Table S1: Number and types of metal restorations in the participants.

## References

[1] Roberts H.W., Berzins D.W., Moore B.K., Charlton D.G. (2009). Metal-ceramic alloys in dentistry: A review. J. Prosthodont..

[2] Arakelyan M., Spagnuolo G., Iaculli F., Dikopova N., Antoshin A., Timashev P., Turkina A. (2022). Minimization of Adverse Effects Associated with Dental Alloys. Materials.

[3] Gaur S., Agnihotri R., Albin S. (2022). Bio-Tribocorrosion of Titanium Dental Implants and Its Toxicological Implications: A Scoping Review. Sci. World J..

[4] Chepelova N., Antoshin A., Voloshin S., Usanova A., Efremov Y., Makeeva M., Evlashin S., Stepanov M., Turkina A., Timashev P. (2023). Oral Galvanism Side Effects: Comparing Alloy Ions and Galvanic Current Effects on the Mucosa-like Model. J. Funct. Biomater..

[5] Mani G., Porter D., Collins S., Schatz T., Ornberg A., Shulfer R. (2024). A review on manufacturing processes of cobalt-chromium alloy implants and its impact on corrosion resistance and biocompatibility. J. Biomed. Mater. Res. B Appl. Biomater..

[6] Zeng L., Xiang N., Wei B. (2014). A comparison of corrosion resistance of cobalt-chromium-molybdenum metal ceramic alloy fabricated with selective laser melting and traditional processing. J. Prosthet. Dent..

[7] Ique M.M.A., Ferreira M.F., Botazzo Delbem A.C., de Mendonça M.R. (2024). Corrosion-induced changes in surface properties and roughness of orthodontic wires. Am. J. Orthod. Dentofac. Orthop..

[8] Bechir F., Bataga S.M., Ungureanu E., Vranceanu D.M., Pacurar M., Bechir E.S., Cotrut C.M. (2021). Experimental Study Regarding the Behavior at Different pH of Two Types of Co-Cr Alloys Used for Prosthetic Restorations. Materials.

[9] Kameda T., Ohkuma K., Oda H., Sano N., Batbayar N., Terashima Y., Sato S., Terada K. (2013). Magnetic fields from electric toothbrushes promote corrosion in orthodontic stainless steel appliances but not in titanium appliances. Dent. Mater. J..

[10] Farrag O.G.A.E.G., Shamaa N.E.-D.A., Elgameay W.E., Bayoumi D.A. (2024). Clinical effect of chlorhexidine and sodium fluoride on corrosion behavior and surface topography of nitinol orthodontic archwires. BMC Oral Health.

[11] Schedle A., Samorapoompichit P., Rausch-Fan X., Franz A., Füreder W., Sperr W., Ellinger A., Slavicek R., Boltz-Nitulescu G., Valent P. (1995). Response of L-929 Fibroblasts, Human Gingival Fibroblasts, and Human Tissue Mast Cells to Various Metal Cations. J. Dent. Res..

[12] Achmad R.B., Auerkari E. (2017). Effects of Chromium on Human Body. Annu. Res. Rev. Biol..

[13] Pavesi T., Moreira J.C. (2020). Mechanisms and individuality in chromium toxicity in humans. J. Appl. Toxicol..

[14] Dotaniya M.L., Saha J.K., Rajendiran S., Coumar M.V., Meena V.D., Kundu S., Patra A.K. (2019). Chromium toxicity mediated by application of chloride and sulfate ions in Vertisol of Central India. Environ. Monit. Assess..

[15] Sun H., Brocato J., Costa M. (2015). Oral Chromium Exposure and Toxicity. Curr. Environ. Health Rep..

[16] Genchi G., Lauria G., Catalano A., Carocci A., Sinicropi M.S. (2023). Prevalence of Cobalt in the Environment and Its Role in Biological Processes. Biology.

[17] Bauer C., Stotter C., Jeyakumar V., Niculescu-Morzsa E., Simlinger B., Rodríguez Ripoll M., Klestil T., Franek F., Nehrer S. (2021). Concentration-Dependent Effects of Cobalt and Chromium Ions on Osteoarthritic Chondrocytes. Cartilage.

[18] Messer R.L., Bishop S., Lucas L.C. (1999). Effects of metallic ion toxicity on human gingival fibroblasts morphology. Biomaterials.

[19] Akbar M., Brewer J.M., Grant M.H. (2011). Effect of chromium and cobalt ions on primary human lymphocytes in vitro. J. Immunotoxicol..

[20] Lan L., Feng Z., Liu X., Zhang B. (2024). The roles of essential trace elements in T cell biology. J. Cell. Mol. Med..

[21] Registration Dossier—ECHA. https://echa.europa.eu/bg/registration-dossier/-/registered-dossier/15506/7/1.

[22] Zhong Q., Pan X., Chen Y., Lian Q., Gao J., Xu Y., Wang J., Shi Z., Cheng H. (2024). Prosthetic Metals: Release, Metabolism and Toxicity. Int. J. Nanomed..

[23] Tomova Z., Tomov D., Vlahova A. (2023). The impact of dental metal restorations on the oral oxidative stress level. J. Clin. Exp. Dent..

[24] Pandey M., Reddy V., Wanjari V. (2019). Comparative evaluation of citric acid and TENS as means for salivary stimulation in adults: An Invivo study. J. Indian Acad. Oral Med. Radiol..

[25] Kim Y.J., Kim Y.K., Kho H.S. (2010). Effects of smoking on trace metal levels in saliva. Oral Dis..

[26] Kassapidou M., Franke Stenport V., Hjalmarsson L., Johansson C.B. (2017). Cobalt-chromium alloys in fixed prosthodontics in Sweden. Acta Biomater. Odontol. Scand..

[27] Tuna S.H., Pekmez N.Ö., Keyf F., Canli F. (2009). The influence of the pure metal components of four different casting alloys on the electrochemical properties of the alloys. Dent. Mater..

[28] Bandyopadhyay A., Traxel K.D., Avila J.D., Mitra I., Bose S. (2020). Biomaterials Science.

[29] Yang L., Zhu Q., Xie X., Cao X., Wu Y., Chen S., Qu J.-E. (2019). Electrochemical behavior of CoCrMo alloy for dental applications in acidic artificial saliva containing albumin. Colloids Surf. B Biointerfaces.

[30] Lu C., Zheng Y., Zhong Q. (2017). Corrosion of dental alloys in artificial saliva with *Streptococcus mutans*. PLoS ONE.

[31] Carek A., Slokar B.L., Bubalo V. (2023). Metal Ions Release from Welded Co-Cr Dental Alloys. Materials.

[32] Sfondrini M.F., Cacciafesta V., Maffia E., Massironi S., Scribante A., Alberti G. (2009). Chromium release from new stainless steel, recycled and nickel-free orthodontic brackets. Angle Orthod..

[33] Brown M.N., Phan L.H., Bryant D.M., Smith R.A., Morrow B.R., Mihalko W.M. (2024). In Vitro Inflammatory Cell-Induced Corrosion Using a Lymphocyte and Macrophage Coculture. J. Arthroplast..

[34] Marti A. (2000). Cobalt-base alloys used in surgery. Injury.

[35] Cheung A.C., Banerjee S., Cherian J.J., Wong F., Butany J., Gilbert C., Overgaard C., Syed K., Zywiel M.G., Jacobs J.J. (2016). Systemic cobalt toxicity from total hip arthroplasties: Review of a rare condition Part 2. measurement, risk factors, and step-wise approach to treatment. Bone Jt. J..

[36] Vaicelyte A., Janssen C., Le Borgne M., Grosgogeat B. (2020). Cobalt–Chromium Dental Alloys: Metal Exposures, Toxicological Risks, CMR Classification, and EU Regulatory Framework. Crystals.

[37] Garhammer P., Hiller K.-A., Reitinger T., Schmalz G. (2004). Metal content of saliva of patients with and without metal restorations. Clin. Oral Investig..

[38] Yassaei S., Dadfarnia S., Ahadian H., Moradi F. (2013). Nickel and chromium levels in the saliva of patients with fixed orthodontic appliances. Orthodontics.

[39] Pan Y., Lin Y., Jiang L., Lin H., Xu C., Lin D., Cheng H. (2020). Removal of dental alloys and titanium attenuates trace metals and biological effects on liver and kidney. Chemosphere.

