# Supplementary material for: Salivary Chromium and Cobalt Concentrations in Patients with Dental Metallic Restorations—A Pilot Study
Source: Dent J (Basel). 2024 Nov 14;12(11):362. doi: 10.3390/dj12110362 (PMC11593078; doi:10.3390/dj12110362)
Supplement: Supplementary file 1 [file dentistry-12-00362-s001.zip › dentistry-3276249-supplementary.pdf]

Table S1. Number and types of metal restorations in the participants.

patient	Number of metal restorations	Amalgam obturations	Crowns				Bridges				
			Number of crowns	Full metal	Metal with vestibular esthetic veneering	Metal-ceramic	Number of bridges	Number of units	Full metal	Metal with vestibular esthetic veneering	Metal-ceramic
1	4	0	3			√	1	3			√
2	3	0	1			√	2	8			√
3	3	0	0				3	9			√
4	2	0	2	√			0	0			
5	3	2	0				1	3		√	
6	3	0	3		√		0	0			
7	3	1	2	√	√		0	0			
8	3	0	2			√	1	4			√
9	3	0	0				3	8			√
10	2	0	0				2	6		√	√
11	2	0	0				2	6			√
12	3	0	2		√	√	1	3			√
13	3	0	3		√	√	0	0			
14	5	0	5			√	0	0			
15	1	0	0				1	7			√
16	2	0	0				2	7			√
17	4	0	2			√	2	8			√
18	3	0	2	√	√		1	6			√
